# The complete mitogenome of the Emerald Ash Borer (EAB), *Agrilus planipennis* (Insecta: Coleoptera: Buprestidae)

**DOI:** 10.1080/23802359.2017.1292476

**Published:** 2017-02-23

**Authors:** Jun Duan, Guoxing Quan, Omprakash Mittapalli, Michel Cusson, Peter J. Krell, Daniel Doucet

**Affiliations:** a Natural Resources Canada, Great Lakes Forestry Centre, Canadian Forest Service, Sault Ste. Marie, Canada;; b Department of Molecular and Cellular Biology, University of Guelph, Guelph, Canada;; c Department of Entomology, Ohio Agricultural Research and Development Center, The Ohio State University, Wooster, OH, USA;; d Natural Resources Canada, Laurentian Forestry Centre, Canadian Forest Service, Québec City, Canada

**Keywords:** EAB, mitochondrial genome, alien invasive pest, jewel beetle

## Abstract

The complete mitogenome of the Emerald Ash Borer (EAB, *Agrilus planipennis*) was obtained by gleaning mitochondrial sequences from whole-genome Illumina sequencing data. The circular genome has 15,942 base pairs and contains 13 protein-coding genes (PCGs), 22 transfer RNAs (tRNAs), 2 ribosomal RNAs (rRNAs) and an A–T-rich region. All PCGs begin with ATN codons. The nucleotide composition is highly asymmetric (31.65% A, 40.25% T, 17.39% G, 10.71% C), with an overall A–T content of 71.9%. Phylogenetic analysis based on insect mitogenomes indicated that EAB is closely related to other Buprestoidea species, clustering most closely with *Chrysochroa fulgidissima*.

The Emerald Ash Borer (EAB), *Agrilus planipennis*, is an invasive phloem-feeding insect pest of North American ash trees originating from East Asia (Herms & McCullough 2014). Since its initial discovery near the cities of Detroit (US) and Windsor (Canada) in 2002, EAB has spread rapidly to other US states and Canadian provinces. Genetic and genomic information are crucial elements to understand the movement of EAB in its introduced range. Likewise, full mitochondrial DNA information from EAB could help resolve the phylogeny of the genus Agrilus, which, with *ca*. 3000 species, is recognized as the most speciose genus in the animal kingdom (Jendek & Poláková 2014).

EABs were collected from infested ash trees in Toledo, Ohio (41°38'59.3"N 83°32'00.9"W). Genomic DNA was sequenced using the paired-end module on Illumina HiSeq2000. Five million pairs of raw reads were randomly chosen for reconstructing the mitogenome. Reads were filtered using Trimmomatic (LEADING: 20, TRAILING: 20, SLIDINGWINDOW: 4:15, Bolger et al. 2014). Quality-trimmed reads longer than 50 bp were used for the mitogenome construction with mitoMaker (http://sourceforge.net/projects/mitomaker/), using that of *T. castaneum* (GenBank accession number: KM009121) as a reference. The mitogenome was annotated using the MITOS web server (Bernt et al. 2013) and coding regions were manually verified by comparison against the NCBI nr database.

The mitogenome of EAB is 15,942 bp long (GenBank accession number: KT363854) and significantly biased towards A and T nucleotides (31.65% A, 40.25% T, 17.39% G, 10.71% C), similar to that of *T. castaneum* (A–T: 71.7%) (Friedrich & Muqim 2003). A total of 37 genes were identified, including 13 protein-coding genes (PCGs), 22 transfer RNAs (tRNAs), and 2 rRNAs. PCGs formed the largest portion (11,331 bp, 71.08%), while rRNAs and tRNAs accounted for 13.26% (2114 bp) and 9.12% (1454 bp), respectively. The A + T-rich region was 1184 bp long, which was located between the tRNA-Ile and 12S rRNA. All 13 PCGs were initiated by typical ATN codons (seven with ATG, four with ATT and two with ATA). Most PCGs ended with TAA codons except for ATP8 and CYTB, which had TAG codons.

Maximum likelihood (ML) analyses were performed to investigate EAB phylogenetic relationships with other coleopterans using the 44 mitogenome sequences available for this order. The 13 PCGs were concatenated and aligned with MUSCLE (Edgar 2004) using default parameters. The ML analysis was carried out using RAxML (Stamatakis 2014), with the optimal substitution model of MeREV, which was selected by Protest3 under the Akaike Information Criterion (AIC). As shown in Figure 1, the phylogenetic analysis placed EAB within the Buprestoidea, and its closest relative is *Chrysochroa fulgidissima*. This complete mitogenome sequence will provide a resource for EAB population genetic research, and may help map routes of EAB invasions.

**Figure 1.**
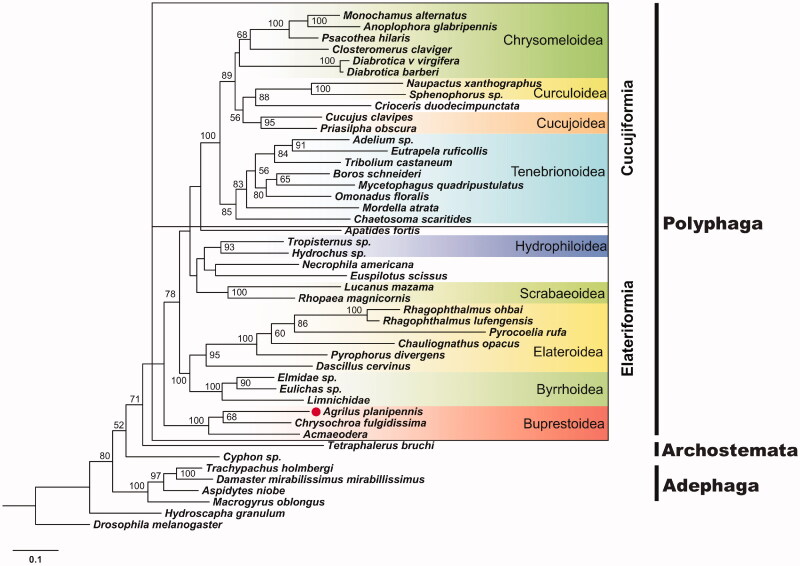
Maximum likelihood estimation of the phylogenetic relationships among EAB and other species within insect order of Coleoptera. Bootstrap values were calculated using 100 bootstrap pseudoreplicates; only values >50 are shown.
